# Emerging therapies in mantle cell lymphoma

**DOI:** 10.1186/s13045-020-00914-1

**Published:** 2020-06-17

**Authors:** Walter Hanel, Narendranath Epperla

**Affiliations:** Division of Hematology, Department of Medicine, The James Cancer Hospital and Solove Research Institute, The Ohio State University, Columbus, OH 43210 USA

**Keywords:** Mantle cell lymphoma, BTK, BCL2, CART, BiTe

## Abstract

Mantle cell lymphoma (MCL) is a rare, B cell non-Hodgkin’s lymphoma with highly heterogeneous clinical presentation and aggressiveness. First-line treatment consists of intensive chemotherapy with autologous stem cell transplant for the fit, transplant eligible patients, or less intensive chemotherapy for the less fit (and transplant-ineligible) patients. Patients eventually relapse with a progressive clinical course. Numerous therapeutic approaches have emerged over the last few years which have significantly changed the treatment landscape of MCL. These therapies consist of targeted approaches such as BTK and BCL2 inhibitors that provide durable therapeutic responses. However, the optimum combination and sequencing of these therapies is unclear and is currently investigated in several ongoing studies. Furthermore, cellular therapies such as chimeric antigen receptor (CAR) T cells and bispecific T cell engager (BiTe) antibodies have shown impressive results and will likely shape treatment approaches in relapsed MCL, especially after failure with BTK inhibitors. Herein, we provide a comprehensive review of past and ongoing studies that will likely significantly impact our approach to MCL treatment in both the frontline (for transplant eligible and ineligible patients) as well as in the relapsed setting. We present the most up to date results from these studies as well as perspectives on future studies in MCL.

## Background

Mantle cell lymphoma (MCL) is a rare, heterogeneous disease comprising around 2.5–6% of B cell non-Hodgkin lymphoma (NHL) [1, 2]. The primary genetic alteration in MCL is the chromosomal translocation (11;14) which leads to CyclinD1 overexpression and uncontrolled cell proliferation. MCL is classified into four morphological variants: leukemic non-nodal, classic, blastoid, and pleomorphic, with the last two considered more aggressive and associated with poorer prognosis [1]. The MCL international prognostic index (MIPI) score stratifies patients based on age, ECOG performance status, LDH, WBC, and Ki-67 positivity into low, intermediate, and prognostic groupings with a 5-year OS of 60%, median OS of 51 months or median OS 29 months, respectively [3]. In addition, TP53 gene mutation at diagnosis is associated with poor response to upfront intensive chemotherapy and poor prognosis [4].

Currently, the approach for the upfront treatment of MCL largely relies on patient-specific factors such as age, overall performance status, and underlying co-morbidities. For the young, transplant eligible patient, treatment generally consists of induction chemotherapy followed by consolidation with an autologous hematopoietic stem cell transplant (auto-HCT) followed by maintenance with rituximab for 3 years. For induction chemotherapy, unlike in diffuse large B cell lymphoma (DLBCL), there is no specific chemotherapy regimen that has been firmly established as the standard of care and the specific regimen used is variable based on the institution or physician practice, although it is generally accepted that the regimen should contain cytarabine. Common regimens include rituximab/dexamethasone/cytarabine/cisplatin (R-DHAP), alternating with rituximab/cyclophosphamide/doxorubicin/vincristine/prednisone (R-CHOP) (R-CHOP/R-DHAP), or rituximab/hyperfractionated cyclophosphamide/vincristine/doxorubicin/dexamethasone alternating with high-dose methotrexate and cytarabine (R-hyperCVAD). For patients unfit for intensive chemotherapy, less toxic chemotherapy treatments are given, such as bendamustine/rituximab (BR) or R-CHOP with or without maintenance rituximab. At disease progression/relapse, targeted agents such as ibrutinib, lenalidomide, bortezomib, or venetoclax are used in succession as monotherapies. Allogeneic HCT (allo-HCT) can provide durable remission in select patients.

The goal of this review is to provide a broad overview of approaches and clinical trials that are currently ongoing in MCL that may lead to novel therapies, some of which may result in significant shifts in the treatment paradigms. Although we will briefly discuss important studies leading to currently approved therapies for MCL, we will reserve much of our focus to ongoing studies in MCL. We will initially begin by reviewing the use of targeted agents including small molecule inhibitors and antibodies in the relapsed/refractory setting and current trials using combinations of these therapies. We will review the exciting new role of cellular therapies in MCL, including chimeric antigen receptor T (CART) cells and bispecific T cell engager (BiTe) antibodies. We will then discuss how targeted therapies currently used in the relapsed setting are being moved to upfront therapy to challenge the paradigm of chemoimmunotherapy as sole therapy in this setting. We will finally discuss how risk-adapted approaches may soon become incorporated into treatment algorithms for MCL.

## Current and future approaches to relapsed/refractory MCL

### Small molecule targeted therapies

#### BTK inhibitors

Bruton’s tyrosine kinase (BTK) is an intracellular kinase downstream from the B cell receptor (BCR) required for normal B cell maturation and BCR-mediated proliferation and survival [5]. BTK signals downstream to activate the ERK, PI3K, NF-kappaB, and NFAT signaling pathways via tyrosine phosphorylation of PLC-gamma2. Many cases of MCL have constitutively active BCR signaling and inhibition of BTK by genetic knockdown results in apoptosis, thus confirming BTK as an important therapeutic target in MCL [6]. Aside from direct cytotoxic effects, inhibition of BTK by ibrutinib may have immune-modulatory effects by increasing peripheral CD4 and CD8 T cells [7]. Currently, several BTK inhibitors have been either FDA approved or are in further development for the treatment of MCL (Table 1).

**Table 1:** BTK inhibitors in the treatment of relapsed/refractory MCL

BTKi	NCT#/publication	Phase	Sample size [median f/up^*^]	Median lines of prior therapy	ORR% [CR%]	Median PFS (months)	% bleeding events [grade ≥ 3]	% A.fib [grade ≥ 3]
Ibrutinib	NCT01236391 [8, 9]	II	111 [26.7]	3	67 [23]	13	59 [5]	6 [5]
Ibrutinib	NCT01646021 [10]	III	139^a^ [20]	2	72 [19]	14.6	10 [8]	4 [4]
Acalabrutinib	NCT02213926 [11–13]	II	124 [15.2]	2	81 [40]	20	33 [2]	0 [0]
Zanubrutinib	NCT02343120 [14]	Ib	43^b^ [10.3]	1	90^c^ [20]	18	30.2 [7]	4.7 [NP]
Zanubrutinib	NCT03206970 [15]	II	86 [9]	2	84 [59]	NR	4.7 [1.2]	0 [0]
LOXO-305	NCT03740529 [16]	I	8 [NP]	3	37.5 [0]	NP	11 [0]	0 [0]
ARQ-531	NCT03162536 [17]	I	1 [NP]	NP	NP	NP	NP	NP

If more than one B cell malignancy was enrolled, responses are for the MCL patients in the trial. Adverse events were for all patients in the trial

Abbreviations: *NR* not reached, *NP* not presented, *Btki* Bruton’s tyrosine kinase inhibitor, *ORR* overall response rate, *CR* complete response, *PFS* progression-free survival, *A.fib* atrial fibrillation

^a^Number enrolled in BTKi arm only

^b^38 relapsed/refractory MCL, 5 patients were treatment naïve MCL

^c^Relapsed/refractory MCL 88.9 [22.2], treatment naïve MCL, 100 [0]

*Median f/up in months

Ibrutinib, a first in class BTK inhibitor, binds covalently to cysteine 481 within the ATP binding domain of BTK resulting in irreversible kinase inhibition. In addition to BTK inhibition, ibrutinib also inhibits interleukin-2 inducible T cell kinase (ITK), tyrosine-protein kinase (TEC), and the epidermal growth factor receptor kinase (EGFR). In the pivotal phase 2 study of relapsed/refractory MCL patients (*n* = 111), ibrutinib demonstrated an overall response rate (ORR) of 67% with a complete response (CR) rate of 23% leading to its FDA approval after at least one prior line of therapy [8]. The median time to response (TTR) in the study was 1.9 months, and duration of response (DOR) was17.5 months. Most common side effects were diarrhea (54%), fatigue (50%), nausea (33%), and dyspnea (32%). Fifty percent of patients experienced a bleeding event (grade ≥ 3, 5%), and 6% experienced atrial fibrillation (grade ≥ 3, 5%). The efficacy of ibrutinib in relapsed MCL was further confirmed in phase III MCL3001 trial in which patients were randomized to either ibrutinib or temsirolimus (*n* = 238 total) [10]. The median PFS was significantly better for patients who received ibrutinib (14.6 months) compared to those who received temsirolimus (6.2 months) (*p* < 0.0001). A pooled analysis of three separate ibrutinib trials (*n* = 370) shown an ORR of 66% (CR rate, 20%), with a median PFS and OS of 12.3 months and 25 months, respectively [18]. When this analysis was restricted to the subgroup of patients receiving ibrutinib as the second line, the survival outcomes were considerably better (median PFS as 28 months and OS was not reached).

Acalabrutinib is a second-generation BTK inhibitor that also binds covalently to cysteine 481 but with low activity towards ITK, TEC, and EGFR [19]. Acalabrutinib demonstrated an ORR of 81% (CR rate of 43%) in a phase II study (ACE-LY-2004, *n* = 124) of relapsed/refractory MCL leading to its FDA approval [11]. At a median follow-up time of 26 months, the median PFS and OS were 20 months and not reached, respectively [11, 12]. The most common side effects included headache (34%), infection (41%), diarrhea (25%), and bleeding (25%). There were only 4% of grade ≥ 3 bleeding events and no events of atrial fibrillation.

Zanubrutinib is another irreversible BTK inhibitor with a similar mechanism of covalent cysteine 481 binding but very low activity towards ITK, TEC, and EGFR [20]. It was recently granted accelerated approval for the treatment of relapsed/refractory MCL based on two phase II studies [15, 21]. Zanubrutinib was found to have an ORR of 84% in each of these studies, but the CR rate was different, with 59% in the BGB-3111-206 study and 22% in the BGB-3111-AU-003 study. The discrepancy may be due to the higher rate of patients with low-risk disease in the BGB-3111-206 study (58% versus 28%) but more importantly due to the differences in response assessment (PET in the BGB-3111-206 versus CT in the BGB-3111-AU-003 study). A pooled analysis of the safety data from 424 patients treated with zanubrutinib showed the most common side effects to be upper respiratory tract infections (23.8%), contusion (17.5%), and diarrhea (14.2%) [22]. Major hemorrhage and atrial fibrillation were seen in 2.1% and 1.9%, respectively, rates lower than that seen with ibrutinib but comparable to acalabrutinib, consistent with their respective kinome profiles.

Although the response rates with single-agent BTK inhibitors are relatively high, approximately one third of patients display primary resistance to BTK inhibitors, and nearly all patients eventually progress (secondary resistance). The outcomes of patients who progress following BTK inhibitors are relatively poor with an ORR ranging between 25 and 42% and median OS between 6 and 10 months with salvage therapies [23–25]. There was no specific advantage of either bendamustine, cytarabine, or lenalidomide when given after progression on ibrutinib [23–25]. Mutational profiling was performed on 15 ibrutinib resistant patients demonstrating a mutation in BTK in only 2 patients (C481S and C481R) and no mutation in PLCG2, thus showing a distinct genetic mechanism of progression compared to the ibrutinib resistant chronic lymphocytic leukemia (CLL) patients [25]. In contrast, p53 mutations were found in 75% of patients while NSD2, a chromatin modifier gene, was mutated in 75% of cases that underwent blastoid transformation, suggesting their significance in ibrutinib resistance. The biology of ibrutinib resistance is currently an active area of investigation, and a full discussion is outside the scope of this review.

Novel BTK inhibitors are currently in clinical development that may have a significant role in the treatment of ibrutinib-resistant disease. LOXO-305 is a highly selective BTK inhibitor, which non-covalently binds to BTK unlike ibrutinib, acalabrutinib, and zanubrutinib that all act by covalent binding. This property gives LOXO-305 higher affinity and selectivity for BTK in addition to inhibition of the C481S mutant, although this is less relevant for MCL as it is for CLL as the C481S mutation rate is much lower in MCL as discussed previously. It is currently being evaluated in a phase 1/2 BRUIN study enrolling patients with relapsed/refractory CLL and MCL with the patients in the MCL group receiving a median of 3 prior lines of therapy at the time of enrollment [26]. Preliminary results showed three responses in 8 patients with MCL, with two of these responders having prior progression on BTK inhibitor therapy, neither of which had a C481S mutation. The most common treatment-emergent adverse events (TEAEs) were fatigue (25%), diarrhea (18%), anemia (14%), and rash (14%). Only 11% of patients had a contusion, with no events of serious bleeding or atrial fibrillation.

ARQ-531 is a reversible multi-kinase inhibitor of not only BTK but Src, Syk, and Fyn, the last two of which have been previously shown to be relevant targets in MCL [27, 28]. Multi-faceted upstream kinase inhibition in the BCR pathway may enhance efficacy over downstream inhibition of BTK alone. ARQ-531 showed better survival compared to ibrutinib in the Eμ-TCL and Eμ-TCL/c-myc transgenic mouse models of CLL and Richter’s transformation, respectively [27]. Furthermore, ARQ-531 was able to inhibit the activation of the C481S BTK mutant as well as PLCγ2 mutants in CLL cells. In a separate study, ARQ-531 was also able to deplete the levels of ERK, Myc, and MCL1 in preclinical models of AML showing an overall highly pleiotropic mechanism of action compared to other BTK inhibitors [29]. Thus, ARQ-531 may have activity against downstream mediators of ibrutinib-resistant MCL. However, further studies are needed specifically within ibrutinib resistant-MCL cells to provide more definitive evidence of its utility within this context.

Preliminary clinical results presented at the 2019 ASH conference show that ARQ-531 has activity in a variety of B cell malignancies that progressed on previous BTK inhibitors [17]. Preliminary safety data during the dose-escalation phase showed that the most common TEAE were nausea (10%), diarrhea (10%), and fatigue (7.5%). There were no atrial fibrillation or grade 3/4 bleeding events noted in the study. As there was only one patient with MCL in this study, its activity in MCL awaits further evaluation in larger studies with more MCL patients. A phase 1b expansion phase at 65 mg daily is currently ongoing in multiple B cell malignancies including MCL.

#### BCL2 inhibitors

The programmed cell death pathway plays an important role in MCL pathogenesis as 90% of MCL cases have been found to overexpress BCL2 [30]. BCL2 may protect MCL cells from apoptogenic signals that would otherwise cause programmed cell death. Venetoclax is a second-generation BH3 mimetic with higher selective inhibition of BCL2 over BCL-XL as compared to the first generation BH3 mimetic navitoclax, thus reducing the off-target effect of thrombocytopenia seen with BCL-XL inhibition while retaining strong BCL2 inhibition. Venetoclax was studied as a single agent in relapsed/refractory B cell NHL patients (*n* = 106) that included 28 patients with MCL [30]. A 3-week ramp-up dosing schedule was used which resulted in only three cases of laboratory tumor lysis syndrome (TLS) and no cases of clinical TLS. For the MCL cohort, the median PFS was 14 months (better than the other NHL subgroups) with a dose of 800 mg sufficient to achieve a durable response. Although not FDA approved for relapsed/refractory MCL, venetoclax is a valuable single-agent option and is currently undergoing investigation in combination therapies (as discussed later).

MCL1, another BCL2 family pro-survival protein, has also been found to be overexpressed in MCL although not at the same high frequency as BCL2, with one study reporting 33% of cases with increased MCL expression on IHC at a cutoff level of > 10% [31]. The MCL1-specific inhibitor MIK665 has shown activity when studied as a single agent in MCL cell lines and patient samples and has shown significant synergism with venetoclax in several PDX models of MCL [32, 33]. MIK665 is currently being studied in phase I clinical trial of relapsed/refractory lymphoma and myeloma (NCT02992483).

#### Proteasome inhibitors

Proteasome inhibition causes MCL cell death through multiple mechanisms, including decreased NF-kappaB signaling by preventing degradation of IκB, cell cycle arrest by inhibiting p27 degradation, and reactive oxygen species generation [34, 35]. Bortezomib is a reversible inhibitor of the 26S proteasome that was the first FDA-approved therapy for relapsed/refractory MCL based on the results from the landmark PINNACLE trial [36, 37].

The second-generation irreversible proteasome inhibitor, carfilzomib induces apoptosis in MCL cell lines and primary samples through inhibition of NF-kappaB and Stat3 signaling [38]. Carfilzomib may have less neurotoxicity compared to bortezomib but at the risk of a higher rate of cardiotoxicity [39]. Carfilzomib was evaluated in phase II clinical trial of relapsed/refractory MCL but suffered from poor accrual with only 4 patients evaluated for treatment response, all of which progressed by three cycles of therapy [40]. Thus, bortezomib is the only proteasome inhibitor currently approved with proven single-agent activity in relapsed/refractory MCL. The potential of this therapeutic class likely resides in its use in combination treatments with other agents.

### Non-cellular immune therapies

#### Lenalidomide

Lenalidomide is a second-generation immunomodulatory agent with superior anti-tumor properties and better side effect profile compared to its analog, thalidomide, and thus has nearly supplanted it in cancer treatment. Lenalidomide showed significant activity in pre-clinical models of MCL through pleiotropic effects including direct cytotoxicity to malignant cells, enhancement of dendritic, NK and T cell activation, inhibition of survival signals provided by stromal support, and suppression of angiogenesis [41–43]. Of note, recent data suggests that angiogenesis is a significant prognostic factor in primary MCL, and SOX11 is an important driver of the angiogenic program, thus providing further importance to this well-established activity of lenalidomide in MCL [44].

Lenalidomide was studied as a single agent in relapsed/refractory NHL in the NHL-002, NHL-003, MCL-001 (EMERGE), and MCL-002 (SPRINT) trials and demonstrated modest single-agent activity in relapsed/refractory MCL in the pre-ibrutinib era [45–49]. The results of these trials have been extensively reviewed previously [50].

#### Monoclonal antibodies

Antibody engineering has been perhaps one of the greatest achievements in cancer therapeutics over the past two decades. The introduction of rituximab brought about significant improvements in responses and OS across all B cell NHL, including MCL. Despite this initial advancement, there has been limited progress even after the development of many uniquely designed antibodies with distinct targets with significant preclinical potential. Several antibodies targeting other markers expressed at a high frequency in MCL including ROR1, CD37, and CD74 are currently being evaluated in MCL.

Obinutuzumab is a glycoengineered type II antibody with increased antibody-dependent cytotoxicity (ADCC) due to enhanced binding to NK cells. Obinutuzumab showed enhanced cell killing of MCL cell lines compared to rituximab [51]. Although, it showed modest single-agent activity when studied in relapsed/refractory MCL (27%); 20% of rituximab refractory patients demonstrated response [52]. Hence, several studies are ongoing using obinutuzumab as a combinatorial approach both in the upfront and relapsed/refractory settings as discussed elsewhere.

ROR1 is an oncoembryonic receptor tyrosine kinase and is only expressed in embryonic and malignant tissues, theoretically providing a much more specific target than the previously discussed surface proteins. It is expressed at a high level in CLL [53, 54] and MCL [55]. It is activated by the ligand Wnt5a and induces proliferation through activation of Rac1 and RhoA [56]. Cirtuzumab (UC-961) is a humanized antibody that targets ROR1 and blocks the binding of Wnt5a and activation of ROR1. Of note, it demonstrated preclinical activity against ibrutinib-resistant MCL [57]. In a study of relapsed/refractory CLL (*n* = 26), 17 among the 22 evaluable patients who received cirtuzumab had stable disease with a median TTNT of 262 days [58]. This led to the phase Ib/II CIRLL trial (NCT03088878) in which cirtuzumab will be studied in combination with ibrutinib. This trial has separate cohorts of CLL and relapsed/refractory MCL. The MCL expansion cohort is currently underway, and preliminary results are eagerly awaited.

CD37 is expressed on normal and malignant B cells. Otlertuzumab is a humanized anti-CD37 antibody, which acts by triggering direct apoptosis by upregulating the pro-apoptotic BCL2 family member, Bim, in target cells. It was studied in a phase I study of patients with relapsed/refractory follicular lymphoma (FL), MCL, and Waldenstrom’s macroglobulinemia [59]. A total of 16 patients were treated, 4 with MCL. Unfortunately, no activity was seen in MCL patients. However, as otlertuzumab was generally well tolerated, rationale combination studies may be feasible to improve on these results. It may be possible that the extent of Bim upregulation was not able to overcome the high rate of BCL2 overexpression seen in MCL and combining otlertuzumab with a BH3 mimetic, such as venetoclax, may offer much better responses. Antibody-drug conjugates (ADC) targeting CD37 is discussed below.

The anti-CD74 antibody milatuzumab showed a promising preclinical activity in MCL, especially when the surface expression of CD74 is enhanced by blocking receptor recycling [60]. In a phase I study where milatuzumab was given as monotherapy in relapsed/refractory NHL (one case of MCL) and CLL, 23 patients were enrolled, and 8 patients had stable disease with no CR or partial responses (PR) [61]. With the high rate of receptor endocytosis, CD74 may be a more attractive target for cytotoxic drug delivery. However, no further trials with milatuzumab or drug conjugated derivatives of milatuzumab are planned at present.

#### Antibody-drug conjugates

ADCs allow targeted delivery of a potent cytotoxic molecule which cannot be otherwise delivered by itself due to toxicity on off-target cells, thus resulting in a much better therapeutic window. Several ADCs have been developed with significant activity in B cell NHL but are still awaiting further studies in MCL.

CD79B is a receptor important in B cell development that is expressed in several subtypes of B cell NHL, including MCL. Polatuzumab vedotin is an anti-CD79B antibody conjugated to the mitotic poison monomethyl auristatin E (MMAE) that works by delivering auristatin E into cells after cleavage of the antibody occurs after endocytosis of the polatuzumab/CD79B complex [62]. It was recently approved in DLBCL in combination with BR [63]. Currently, polatuzumab is being studied in several trials both in frontline and relapsed settings as a combinatorial approach (with chemotherapy or other novel agents) in DLBCL and FL. As of yet, no MCL specific trials are currently registered.

CD22 is an inhibitory component of the B cell receptor expressed early in B cell development at the pre-B cell stage as well as mature B lymphocytes but is lost upon differentiation into plasma cells. The expression of CD22 is nearly universal in MCL. Several ADCs targeting CD22 have been developed. Inotuzumab ozogamicin is a humanized IgG4 antibody targeting CD22 which is conjugated to the cytotoxic agent calicheamicin [64]. When calicheamicin is internalized, it binds to DNA causing double-stranded breaks and apoptosis. Inotuzumab is FDA-approved for the treatment of B cell precursor ALL. Although early phase clinical studies in relapsed/refractory B cell NHL have shown promise, a phase III study was discontinued early due to lack of efficacy [65]. Pinatuzumab vedotin is another anti-CD22 ADC that is conjugated to MMAE leading to microtubule inhibition upon internalization. In the phase II ROMULUS study, patients with relapsed/refractory DLBCL and FL were randomized to either rituximab plus pinatuzumab or rituximab plus polatuzumab [66]. Although both pinatuzumab + rituximab and polatuzumab + rituximab were associated with similar ORR (60% vs 54%) and CR (26% vs 21%), polatuzumab was associated with a better duration of response (13.4 months vs 6.2 months) and OS (20.1 months vs 16.5 months). Thus, pinatuzumab was dropped from further development in favor of polatuzumab. Trph-222 is an anti-CD22 antibody conjugated to maytansine, a microtubule targeting compound, using an optimized site-specific protein modification technology [67]. It is currently in a phase I study in relapsed/refractory B cell lymphomas (NCT03682796).

Similar to CART cells and BiTe antibodies (discussed later), ADCs that target the ubiquitous B-lineage surface marker CD19 have been developed. Loncastuximab tesirine (ADCT-402) is a humanized anti-CD19 IgG1 conjugated to pyrrolobenzodiazepine (PDB) which is a DNA damaging agent. In a large phase I study (*n* = 88) of patients with relapsed/refractory B cell NHL, ADCT-402, (at doses ≥ 120 ug/kg) demonstrated an ORR of 59.4% (CR rate = 40.6%) [68]. Of the 9 patients with MCL (all doses), an ORR was 44.4% and CR rate was 33.3%, respectively. ADCT-402 was overall tolerable with the most common TEAE (≥ 20%) being hematologic abnormalities, fatigue, edema, LFT abnormalities, fatigue, and dyspnea. These results led to three phase II clinical trials that are currently ongoing: ADCT-402 monotherapy in relapsed/refractory DLBCL (NCT03589469), ADCT-402 in combination with ibrutinib in relapsed/refractory DLBCL and MCL, and ADCT-402 in combination with durvalumab in relapsed/refractory DLBCL, MCL, and FL (NCT036855344). Two other anti-CD19 ADCs, coltaximab ravtansine and denintuzumab, were investigated but were not pursued further either due to limited efficacy or safety concerns [69–71].

Naratuximab emtansine (IMGN529) is an ADC linked to the maytansinoid DM1 which targets CD37 [72]. An initial phase I study shown a tolerable safety profile in relapsed/refractory B cell NHL [73]. It is currently being evaluated in a phase II trial in patients with relapsed/refractory B cell NHL in combination with rituximab (NCT02564744). Another anti-CD37 ADC, AGS67E, is also in clinical development. AGS67E is a human IgG2 antibody conjugated to MMAE. In a phase I trial of 50 patients with B and T cell NHL (only 2 patients with MCL), it demonstrated an ORR of 22% (CR rate = 11%) [74]. Expansion cohorts of DLBCL and cutaneous T cell lymphoma are ongoing.

The BTK inhibitors are now used extensively as a first-line treatment of relapsed/refractory MCL and the biology of BTK resistant MCL is quite different from BTK sensitive MCL, it is unclear if the response rates found in the earlier trials of bortezomib, lenalidomide, and the various antibody therapies can be extrapolated to the BTK resistant patients. Overall, it is highly unlikely that single-agent therapy (aside from cellular therapies, discussed later) will provide durable responses in this population and combination strategies will be needed to provide more meaningful responses.

### Combination therapy

#### Combinations of individually active agents

Several past and ongoing single-arm studies are evaluating if the combination of therapies already known to have single-agent activity in MCL discussed above can prolong the PFS benefit of either agent alone (Table 2). While we may not be able to review every one of these trials, we will review those that have either excellent preclinical rationale or promising preliminary results. It is important to interpret these studies very carefully, especially when based on PFS alone, as it is difficult to conclude that combination therapy is more effective than their sequential use without a randomized comparison of these two approaches. This may be of less importance in BTK inhibitor combination studies given the overall lack of durable responses with therapies used after BTK resistance as discussed previously. Nonetheless, these studies will provide preliminary evidence for larger, well-designed randomized clinical trials to confirm that combinations of active agents should become the standard in place of single-agent use.

**Table 2:** Investigative combination treatments for relapsed/refractory MCL

Combination therapy	NCT#/publication	Phase	Sample size [median f/u^*^]	Median lines of prior therapy	ORR% [CR%]	Median PFS (months)	Grade ≥ 3 (%)^**a**^
Ibrutinib/rituximab	NCT01880567 [75]	II	50 [16.5]	3	88 [44]	NR	A.fib (12), renal and urinary disorder (6)
Ibrutinib/ venetoclax	NCT02471391 [76, 77]	II	23 [37.5]	2	71 [62]	29	Diarrhea (12), soft tissue infection (8), lower respiratory tract infection (8) TLS (8), a.fib (8)
Ibrutinib/ venetoclax	NCT03112174	III	ongoing				
Obinutuzumab/ibrutinib	NCT02558816 [78]	I	9 [23.5]	1	87 [87]	NR	none
Obinutuzumab/ibrutinib/ venetoclax	NCT02558816 [78]	I	12 [6.5]	2	66.6 [25]	NR	none
Lenalidomide/rituximab	NCT00294632 [79]	II	44 [23.1]	2	57 [36]	11.1	Fatigue (14), non-neutropenic infection (7), hypercalcemia (7), hyperuricemia (7)
Lenalidomide/rituximab^b^	NCT00783367 [80]	II	11 [39.2]	3	55 [55]	24.4	Hypokalemia (10), hypophosphatemia (6)
Lenalidomide/obinutuzumab	NCT01582776	II	13 [14.5]	2	46.2 [15.4]	NP	Infections (12.5)
Ibrutinib/lenalidomide/ rituximab	NCT02460276 [81]	II	50 [17.8]	2	76 [56]	16	Infections (26), rash (14), GI (12), vascular (10)
Palbociclib/ibrutinib	NCT02159755 [82]	I	27 [25.6]	1	67 [37]	NP	Hypertension (15), febrile neutropenia (15), lung infection (11), URI (7), fatigue (7), transaminitis (7) rash (7)
Palbociclib/ibrutinib	NCT03478514	II	ongoing				

Abbreviations: *NR* not reached, *NP* not presented, *ORR* overall response rate, *CR* complete response, *PFS* progression-free survival, *A.fib* atrial fibrillation

^a^Non-hematologic grade ≥ 3 AE in > 5% of patients

^b^Rituximab refractory

*Median f/up in months

The combination of ibrutinib and venetoclax has shown synergism in preclinical models of MCL with enhanced levels of dephosphorylation of substrates within the BTK pathway as well as a reduction in levels of anti-apoptotic BCL2 family members [83]. A phase II study (AIM study) was conducted to evaluate this combination in relapsed/refractory MCL [76]. This study (*n* = 24) had an initial ibrutinib lead-in phase for one cycle followed by venetoclax ramp-up to 400 mg. The CR rate was 42% and 62% (*n* = 23 with relapsed/refractory MCL) based on CT and PET, respectively. Results of minimal residual disease (MRD) assessment showed a negativity frequency of 67% of the bone marrow by flow cytometry and 38% in the blood by PCR [76]. An amendment to the study allowed people to discontinue therapy if they achieved MRD negativity. Updated analysis of this study was presented at the ASH 2019 conference, which showed a median PFS and OS of 29 months and 32 months, respectively, at a median follow-up of 37.5 months [77]. Of note, the median OS was increased compared to that reported for the single-agent ibrutinib study at 22.5 months [9]. Fifty percent of patients who harbored TP53 mutation (six of 12) responded, and among these, five had a response that extended out to a least 24 months. Of the five patients who obtained MRD negativity, four patients remained free of clinical or MRD progression while still being off therapy at the time of last follow-up (6, 13, 17, and 18 months). Thus, ibrutinib/venetoclax combination therapy may offer deeper and more durable responses to patients compared to monotherapy alone, even in the presence of TP53 mutations. Even more intriguing is the possibility of a limited course of therapy of ibrutinib/venetoclax to induce long-term durable remissions in certain patients. The phase III SYMPATICO study (NCT03112174), a randomized study of ibrutinib/venetoclax versus ibrutinib alone, is currently ongoing. This study will establish whether ibrutinib should be given along with venetoclax or if venetoclax should be reserved until after progression while on ibrutinib.

Obinutuzumab counteracts venetoclax resistance by the reduction in NF-kappaB signaling leading to a reduction in BCL-XL levels in primary MCL cells [84]. This in addition to the known synergistic activity of ibrutinib and venetoclax as discussed above provides a significant rationale for the combination of obinutuzumab/ibrutinib/venetoclax which is currently being evaluated in the ongoing OASIS study [85]. This non-randomized study has three separate recruitment steps: obinutuzumab with ibrutinib in relapsed/refractory MCL (step A), obinutuzumab with ibrutinib and venetoclax in relapsed/refractory MCL (step B), and obinutuzumab with ibrutinib and venetoclax in newly diagnosed MCL (step C, discussed below). For steps B and C, venetoclax was added at cycle 2 with weekly ramp-up. In the relapsed/refractory MCL, both combinations were well tolerated with DLT not reached up to 800 mg of venetoclax. In step A (*n* = 9, 2 with blastoid MCL), 87% of patients were in CR and 67% (4 of 6) of patients were MRD negative by PCR in the blood and bone marrow after cycle 6. For step B (*n* = 12, 4 with blastoid MCL), four patients had progressive disease during the first two cycles. Of nine patients assessed after cycle 6, five were in CR and the MRD analysis is pending. These preliminary results established obinutuzumab/venetoclax/ibrutinib combination to be safe and these responses are promising, but larger studies will be needed to define if this combination provides better efficacy compared to the individual drugs or two-drug combinations in relapsed/refractory MCL.

As lenalidomide has potent immunomodulatory activity with the ability to activate antigen-presenting cells (APCs), there is a significant pre-clinical rationale for combinations with either rituximab or obinutuzumab to enhance the ADCC induced by these antibodies. Aside from this, lenalidomide may re-sensitize rituximab resistant cells. In a phase 1/2 study of relapsed/refractory MCL patients, lenalidomide/rituximab demonstrated an ORR of 57% and a CR rate of 36%, which were higher than lenalidomide monotherapy [79]. In another study, lenalidomide was given initially as a lead-in therapy for 2 months followed by concurrent lenalidomide/rituximab in rituximab refractory patients. The study aimed to test whether lenalidomide could re-sensitize the disease to rituximab [80]. In the 11 patients with MCL, an ORR of 55% (CR rate = 36%) was seen after lenalidomide with no further responses after the addition of rituximab but with the 2 PR improving to CR to 55% [80]. Lenalidomide/rituximab was further combined with ibrutinib in the Nordic phase 1/2 PHILEMON study with relapsed/refractory MCL (*n* = 50) [81]. With a median follow-up of 17.8 months, ORR was 76% with a CR rate of 56%. The lenalidomide/obinutuzumab was studied in relapsed/refractory B cell NHL, which included 13 patients with MCL and demonstrated an ORR of 39% and a CR rate of 23%.

Although combination chemotherapy for second-line therapy is generally falling out of favor to more targeted therapies or clinical trial enrollment, there is one chemotherapy regimen worthy of mention in the relapsed/refractory setting. In a retrospective review of patients following progression on ibrutinib, R-BAC (rituximab/bendamustine/cytarabine) had an ORR of 83% (CR rate of 60%) with a median PFS and OS of 10.1 months and 12.5 months, respectively [86]. Notably, 31% of patients were able to bridge to allo-HCT. Although responses were not durable, R-BAC may be a potentially valuable option for patients in need of cytoreduction before CART cell therapy or allo-HCT, especially in the setting of ibrutinib failure which often has a rapidly progressive course.

### Combination of active therapies with investigational agents

Several investigational drugs have shown little or no clinical activity in MCL by themselves. However, strong preclinical evidence and rationale have led to clinical trials evaluating their use in combination with active therapies. A few notable ongoing clinical trials are discussed below.

#### CDK4/6 inhibitors

The *t*(11:14) is nearly universal in MCL and places cyclinD1 into the immunoglobulin locus resulting in overexpression of cyclinD1, thus leading to phosphorylation of retinoblastoma by CDK4/6 resulting in its inactivation and transition through the G1/S checkpoint. Thus, CDK inhibitors naturally arose as an extremely attractive rationale therapy for MCL. Interestingly, there is a preferential expression of CDK4 relative to CDK6 in MCL cells [87]. Of the specific CDK inhibitors currently, FDA approved, the CDK4 selective inhibitor palbociclib may have a more selective therapeutic benefit in MCL. In a phase 1b study of relapsed/refractory MCL, palbociclib given as monotherapy (*n* = 17) demonstrated an ORR of 18% with 41% of patients having stable disease [88]. In patients who had a response, the median DOR was 18 months, suggesting a subset of MCL patients with high sensitivity to CDK4 inhibition. Pre-clinical studies on MCL cells showed that prolonged G1 arrest by palbociclib leads to the induction of the PI3K inhibitor, PIK3IP1, thus preventing downstream activation of the PI3K pathway from BTK, an important mechanism of ibrutinib resistance [89]. This led to a phase 1 study evaluating the safety of the palbociclib/ibrutinib combination in relapsed/refractory MCL [82]. In the study, the combination was tolerable with cytopenias (neutropenia [41%] and thrombocytopenia [30%]), febrile neutropenia (15%), hypertension (15%), and lung infection (11%) being the most common grade 3/4 toxicities The ORR and CR rate were 67% and 37%, respectively, with a 2 year PFS of 59.4%. Currently, there is an ongoing phase II trial using this combination in relapsed/refractory MCL (NCT02159755). Current or past clinical trials of other CDK inhibitors have been recently reviewed. Overall, the single-agent activity of these have been relatively mild and will likely require combination studies with other agents [87]. However, one particular mention is voruciclib, a broad CDK inhibitor (with activity to CDK1, 4, 6, in addition to CDK9), which is currently undergoing evaluation in early phase studies as monotherapy in B cell malignancies (NCT03547115). This drug showed increased activity with venetoclax in preclinical studies of DLBCL [90]. Thus, further studies of this combination in MCL is warranted.

### Cellular immune therapies

#### CAR-T cell therapy

CART therapy revolutionized the treatment landscape of relapsed/refractory DLBCL and B cell acute lymphoblastic leukemia (ALL) by providing durable responses to patients that had otherwise incurable disease. In the ZUMA-2 trial, the KTE-X19 product (axicabtagene ciloleucel) was tested in relapsed/refractory MCL patients [91]. Inclusion criteria included the failure of prior chemotherapy and anti-CD20 antibody therapy, and at least prior use of a BTK inhibitor, although a failure of BTK inhibitor therapy was not a requirement for enrollment. Bridging therapy was allowed as needed for disease stability during the manufacturing process with either steroids or a BTK inhibitor. In the intent to treat population (*n* = 74), the ORR was 85% (CR rate was 59%) with a median TTR of 1 month. At a median follow up of 12.3 months, 57% of patients were in remission with a 12-month PFS and OS of 61% and 83%, respectively. There was no specific risk factor that predicted a lack of response to therapy, although the numbers in the subgroup analysis were small. The rate of cytokine release syndrome (CRS) was 91% (15% grade ≥ 3), and 63% had neurological events (31% grade ≥ 3). Grade ≥ 3 infections occurred in 32% of patients, and 26% of patients had grade 3 or higher cytopenias after 90 days from the infusion. Overall, the side effect profile was relatively similar to previously reported with prior CART trials [92, 93]. Given the impressive results on this primary efficacy analysis, KTE-X19 is currently undergoing fast-track review by the FDA.

#### BiTe antibodies

BiTes are comprised of two distinct antibody chain combinations each able to recognize a different epitope, one with specificity for an epitope present on T cells and the other of which recognizes an epitope on the target cell of interest resulting in direct cell-mediated toxicity of the tumor cell [94]. Four BiTes have shown promising activity in relapsed/refractory B cell NHL, including MCL (Table 3).

**Table 3:** BiTes currently in trials

BiTe	NCT#/publication	Route/administration schedule	Phase	Sample size* [follow-up**]	Median lines of prior therapy	ORR% [CR%]	CRS [grade ≥ 3]	Neurotoxicity [grade ≥ 3]
Blinatumomab	NCT00274742 [95, 96]	IV continuous infusion over 4 or 8 weeks	I	24 [5.2]	3	71.1^a^ [42.8]	NP [NP]	71 [22]
Mosunetuzumab	NCT02500407 [97]	IV once every 21 days	I/Ib	23 [NP]	3	NP [NP]	28.9 [1.4]	43.7 [3.2]
REGN1979	NCT03888105 [98]	IV weekly for 12 weeks, then every 2 weeks for 24 weeks	I	6 [NP]	3	NP [NP]	57 [7.2]	NP [3.1]
GEN3013	NCT03625037 [99]	Subcutaneous weekly: cycle 1–2; every 2 weeks cycle 3-6; every 28 days thereafter	I/II	NP	3	NP [NP]	50 [0]	0 [0]

Abbreviations: *NR* not reached, *NP* not presented, *ORR* overall response rate, *CR* complete response, *PFS* progression-free survival, *CRS* cytokine release syndrome

^a^Response rate at the target dose of ≥ 60 ug/m^2^/day (*n* = 7 MCL patients)

*The sample size denotes only MCL patients

**f/up in months

The most advanced BiTe in clinical development for B cell malignancies is blinatumomab, an antibody that crosslinks B cells and T cells by ligating CD3 and CD19. In phase I trial of relapsed/refractory NHL (*n* = 76), blinatumomab demonstrated an ORR of 69% and a CR rate of 37% [95] with durable responses in some patients on long-term follow-up [96]. Of note, patients with relapsed/refractory MCL (*n* = 24) had a higher ORR compared to DLBCL (71% versus 55%, respectively) [95]. The most significant toxicity was neurological events with 13 patients discontinuing treatment due to grade 3 or higher events. Blinatumomab is currently undergoing evaluation in combination with other therapies (NCT02811679, NCT03072771, NCT02568553, NCT03340766, and NCT03605589).

Mosunetuzumab is another BiTe in clinical development for B-NHL that ligates CD3 and CD20. Results of the ongoing phase 1/1b dose-escalation study were presented at the 2019 ASH conference [97]. The trial enrolled 270 patients, including 23 patients with MCL. Of note, 30 patients had prior CART therapy. Mosunetuzumab was given once every 21 days (after initial weekly dosing during the first cycle) for 8 cycles, with the continuation of therapy for patients with a PR or stable disease after the 8 cycles to a maximum of 17 cycles. CRS occurred in 28.9% of patients (*n* = 3, grade ≥ 3) and neurotoxicity in 43.7% of patients (*n* = 1, grade ≥ 3). Among 124 evaluable patients, ORR was 37.1% with a CR rate of 19.4%, with 17 patients (13.7%) remaining in CR after 16 months of treatment. MCL-specific response rates were not reported. In patients that had prior CART therapy, the ORR was 38.9% (CR rate = 22%). Thus, mosunetuzumab demonstrated activity in a heavily pretreated population with a lower rate of CRS or neurotoxicity seen previously with CART or blinatumomab. These results are encouraging, and further studies with combination treatments may prolong the durability of responses.

REGN1979, a CD20/CD3 BiTe, is an IgG4 antibody that is modified to reduce binding to the Fc receptor has been studied in relapsed/refractory B cell NHL. The results of the phase I study were recently reported at the 2019 ASH conference [98]. REGN1979 was given every week for a total of 12 weeks followed by biweekly dosing for 12 more doses. Ninety-six patients (6 with MCL) were enrolled, 12 patients with prior CART. The CRS rate was 57% (*n* = 7 with grade ≥ 3). Grade 3 or higher neurotoxicity occurred in two patients. The trial was suspended temporarily due to a patient’s death from TLS for the protocol amendment. Responses were evaluated over a broad range of dosages with dosage-dependent responses seen. With treatment ≥ 80 mg, the DLBCL cohort demonstrated an ORR of 57.9% (CR rate = 42.1%), with CRs seen in post CART patients, while the FL cohort demonstrated an ORR of 95.5% (CR rate = 77.3%) with ≥ 5 mg. These impressive response rates in heavily pre-treated patients may be at a tradeoff with the higher CRS rate seen with this BiTe compared to mosunetuzumab, but larger patient numbers treated at the RP2D will be needed for a more accurate comparison with the other BiTes. A global phase II study is currently planned enrolling relapsed/refractory NHLs including MCL.

GEN3013, a CD20/CD3 BiTe, is an IgG1 antibody that is unique in that it is administered subcutaneously rather than IV [100]. In pre-clinical models, subcutaneous administration demonstrated similar bioavailability and B cell depletion as IV administration, but with lower plasma cytokine levels and was hypothesized to result in less CRS but with the same responses in patients [100]. The preliminary results of a dose-escalation study on 18 patients with relapsed/refractory NHL (*n* = 14 with DLBCL), were presented at the 2019 ASH conference [99]. Patients were treated weekly for two 28-day cycles followed by every 2 weeks for 4 cycles, then monthly until toxicity or progression. The CRS rate was 50% but none with grade ≥ 3 and no patients had neurologic symptoms. There was a patient with a CR at a dose of 120 μg, with no DLTs yet with escalation still ongoing. A phase II trial is planned once the recommended phase II dosage RP2D is found.

## Current approaches to the upfront treatment of MCL

A vast majority of the current studies that aim to improve upfront therapy in the fit patient involve the incorporation of targeted agents already known to have activity in the relapsed/refractory. These agents include ibrutinib, lenalidomide, or bortezomib that are incorporated into either the induction phase, maintenance phase, or both phases of treatment (Table 4). One of the main goals of these approaches is to deepen the responses achieved with chemotherapy and thereby achieving more durable remissions. This is extremely important in the younger MCL patient as the relapsed disease becomes much harder to treat with a lower chance of durable response with available therapies aside from allogeneic SCT. Another important goal is to decrease or eliminate the amount of cytotoxic chemotherapy administered upfront without compromising the response rates and long-term outcomes. In this section, we will first review the past and ongoing upfront novel treatment approaches in the young, fit patient followed by the older, transplant-ineligible patient. We will then discuss the exciting new approach of risk-adapted therapy particularly as it relates to the upfront treatment of MCL.

**Table 4:** Investigative front line treatments for the newly diagnosed transplant eligible MCL patients

Therapy	NCT#/publication	Phase	Sample size [follow-up^*^]	ORR% [CR%]	Median PFS	Grade ≥ 3 (%)^**a**^
R-HyperCVAD+ bortezomib	[101]	II	95 [44]	100 [82]	55	Neutropenic fever (9)
v-BEAM	[102]	I/II	23 [58.5]	95 [86]^b^	NR	Neutropenic fever (59), anorexia (21), peripheral neuropathy (19), orthostatic hypotension (16), ileus (9)
Maintenance bortezomib days 1, 4, 8, 11 of 21 days × 4 cycles vs. maintenance bortezomib weekly for 4 weeks on /4 weeks off × 9 cycles	NCT00310037 [103]	III	151^c^ [96]	–	106.8 v NR	NP^d^
Maintenance bortezomib every 2 weeks × 2 years vs observation	[104, 105]	III	135^e^ [77.5]	–	NR v NR	Infections (7)
Lenalidomide + R-CHOP → R-HIDAC → R2	NCT02633137	II	ongoing			
Maintenance R2 vs rituximab	NCT02354313	III	ongoing			
R2	NCT01472562 [106, 107]	II	38 [64]	92 [64]	NR	Infections (19.4), tumor flare (11), abdominal pain (5), serum sickness (5), syncope (5), neutropenic fever (5)
(R-CHOP/R-DHAP → auto-HCT) vs (R-CHOP/R-DHAP + ibrutinib → auto-HCT → ibrutinib) vs (R-CHOP/R-DHAP + ibrutinib → ibrutinib)	NCT02858258 [108]	III	ongoing			
Acalabrutinib + BR/R-HiDAC → auto-HCT	NCT03623373	II	ongoing			
Ibrutinib + Rituximab → R-hyperCVAD	NCT02427620 [109]	II	131 [22]	100 [94]^f^	NR	Fatigue (8), myalgia (8), rash (8)^g^

Abbreviations: *NR* not reached, *NP* not presented, *ORR* overall response rate, *CR* complete response, *PFS* progression-free survival, *auto-HCT* autologous hematopoietic cell transplantation, *R2* lenalidomide (Revlimid) and rituximab

^a^Non-hematologic grade ≥ 3 AE in > 5% of patients

^b^Response measured at 100 days post-transplant

^c^Number of patients enrolled start of induction, 50 patients were randomized to a twice-weekly schedule and 52 patients to a weekly schedule

^d^Specific toxicities were not presented, but 19 patients withdrew from the study due to AE (28% of patients in a twice-weekly schedule and 13% in the weekly schedule) including 4 treatment-related deaths

^e^44% of patients initially enrolled went on to randomization

^f^Response rate after completing both parts of the treatment. The ORR% after completing ibrutinib + rituximab was 95

^g^AE reported for the ibrutinib + rituximab part of therapy

*f/up in months

### Upfront treatment approaches for the transplant eligible patient

#### Bortezomib in frontline therapy

Incorporation of bortezomib into the upfront setting has been studied extensively in multiple trials as part of induction chemotherapy, as part of the auto-HCT conditioning regimen, and as maintenance therapy following auto-HCT. A phase II study evaluated the outcomes following the addition of bortezomib during both alternating courses of the hyper-CVAD regimen [101]. After a follow-up of 44 months, the median time to treatment failure (TTF) was 55 months, which was comparable to the median TTF of hyper-CVAD alone at 56.4 months from a previous study, thus showing no improvement in the long-term outcomes of the addition of bortezomib to hyperCVAD in the upfront setting.

Incorporation of bortezomib into the BCNU/cytarabine/etoposide/melphalan (BEAM) preparative regimen (V-BEAM) before auto-HCT was studied in a phase 1/2 study of B cell NHL patients (*n* = 42) with most MCL being the most common histology (*n* = 23) [102]. The PFS was not significantly different among the MCL patients who received V-BEAM relative to BEAM (historical control) in CR1; however, there was an increased incidence of autonomic dysfunction and ileus noted in the study.

Two separate trials looked at the benefit of bortezomib in maintenance therapy. The phase II CALGB 50403 studied two different bortezomib dosing strategies in a randomized fashion in the maintenance setting following induction chemotherapy and auto-HCT [110]. One arm received bortezomib at 3 mg/m^2^ IV on days 1, 3, 8, and 11 of a 21-day cycle for 4 cycles, and the other arm received 6 mg/m^2^ IV four times weekly every 8 weeks for 18 months. At the 8-year follow-up, there was no PFS benefit in those receiving bortezomib maintenance compared to the historical control data (CALGB 59909); however, a PFS benefit could be seen when comparing these two populations from the time of transplant [103]. In addition, PFS was not significantly different between the maintenance and consolidative arms of the CALGB 50403 trial [103]. In the European MCL HAVON 75 MCL trial, patients were randomized to either no further treatment or bortezomib 1.3 mg/m^2^ given IV every 2 weeks for 2 years following induction chemotherapy and auto-HCT. There was no significant difference in the EFS or OS at 5 years between the two groups [104, 105]. Thus, taking these studies together, there is currently no clearly defined role of bortezomib in the upfront treatment of the young fit patient with MCL.

#### Lenalidomide in frontline therapy

Unlike bortezomib, lenalidomide is more difficult to administer with aggressive chemotherapy regimens due to the overlapping myelotoxicity of these therapies. Although most studies have incorporated lenalidomide with less myelosuppressive regimens in older patients, there have been some trials of lenalidomide in combination with chemotherapy in the upfront therapy in younger patients. An ongoing single institutional phase II trial (NCT02633137) incorporates lenalidomide into both induction and maintenance by initially giving lenalidomide with R-CHOP (Len-R-CHOP) for 4 cycles followed by consolidation with 2 cycles of rituximab and high-dose ara-C (R-HiDAC) followed by a maintenance phase of lenalidomide and rituximab (R2) for a total of 6 cycles. Of note, this study does not include an auto-HCT and thus reduces the overall intensity and amount of chemotherapy delivered.

Given the overall comparable efficacy of R2 as compared to combination chemotherapy in FL in the RELEVANCE trial [111] and its activity in relapsed/refractory MCL [79, 80], this combination was studied in an upfront phase II trial which included young patients with low or intermediate risk MIPI scores and unfit patients with higher MIPI scores [106, 112]. Both induction and maintenance phases were included in this trial, and patients received R2 therapy until disease progression. At a median follow-up of 64 months (*n* = 38), the 3-year PFS and OS were 80% and 90%, respectively [106]. Thus, R2 may be a potential option for the young patient with a less aggressive disease but a larger randomized study with a direct comparator arm is needed within this patient subset to make conclusions that are more firm on upfront R2 in treatment in MCL. The relative benefit of R2 compared to rituximab alone in the maintenance setting after induction chemotherapy, and auto-HCT is currently undergoing evaluation in the Italian phase III MCL0208 study (NCT02354313).

#### BTK inhibitors in frontline therapy

Given the significant activity of BTK inhibition in relapsed/refractory MCL, approaches trying to incorporate ibrutinib into the front line setting are ongoing. The TRIANGLE study is a large phase III study by the European MCL network for young (age < 65), transplant eligible MCL patients, which incorporates ibrutinib in induction and maintenance therapy [108]. In the study, the patients were randomized into three separate arms: one arm received induction chemotherapy (R-CHOP alternating with R-DHAP for 6 cycles) followed by auto-HCT (either BEAM or THAM conditioning, randomized by site), a second arm received the same regimen as arm 1 with the addition of ibrutinib during induction (only to R-CHOP cycles on days 1-19) and maintenance therapy (560 mg for 2 years), and the final arm received the same regimen as in the second arm but these patients will not undergo an auto-HCT. Rituximab could be added to any of these arms at the discretion of the treating physician. The primary outcome was the investigator-assessed failure-free survival (FFS). MRD assessment was also incorporated into the trial as an exploratory endpoint but was not used to guide treatment decisions as in ECOG 4151 (discussed below). As of July 2019, 511 patients have been randomized. In the completed safety run-in of the initial 50 patients, the feasibility of the two experimental arms was confirmed with no major differences in hematological and other toxicities and no major delays during induction. This trial will ultimately address whether incorporating ibrutinib into frontline chemotherapy provides a more durable response in MCL and whether auto-HCT adds any further benefit if ibrutinib is incorporated into frontline treatment. One U.S. group is evaluating the addition of acalabrutinib to the front line setting in transplant eligible patients by incorporating acalabrutinib into BR and alternating this with cycles of cytarabine and rituximab as induction chemotherapy before transplant (NCT03623373).

The MD Anderson group is currently pursuing a distinct chemo-sparing approach utilizing ibrutinib in the upfront treatment. In a phase II study (NCT02427620), ibrutinib was initially given with rituximab (part A) instead of concurrently with chemotherapy in newly diagnosed MCL patients until disease progression or an overall response was achieved. This was followed by consolidation with R-hyperCVAD/R-MTX without ibrutinib (part B) for 4 cycles. In the preliminary analysis presented at the 2019 malignant lymphoma meeting in Lugano, the ORR (*n* = 50) was 100% (CR rate = 90%) after a median of 6 cycles of part A [109]. MRD assessment of the bone marrow by flow cytometry at the time of best response showed an impressive negativity rate of 91%. Few patients had grade 3–4 toxicities during part A (4% myelosuppression and 8% fatigue). At a median follow-up of 36 months, 3-year PFS and OS were 89% and 100%, respectively. These results show that ibrutinib with rituximab is highly active in the treatment of naïve patient with the potential to reduce the amount of cytotoxic chemotherapy delivered in the frontline setting.

### Novel upfront approaches for the transplant-ineligible patient

Just as in the transplant-eligible patient, several trials are incorporating newer targeted agents into the upfront setting in transplant eligible patients (Table 5).

**Table 5:** Investigative front line treatment for the newly diagnosed transplant-ineligible MCL patients

Therapy	NCT#/publication	Phase	Sample size [follow-up^*^]	ORR% [CR%]	Median PFS (mos)	Grade ≥ 3 (%)
BR + lenalidomide → lenalidomide	NCT00963534 [113]	I/II	51 [31]	80 [64]	42	Infection (42), rash (18), allergic reaction (12), mucositis (6), musculoskeletal pain (6), anorexia (6)
(R-CHOP/R-HAD X 4 vs R-CHOP X 8) ≥ (R2 vs rituximab)	NCT01865110	III	Ongoing			
(BR vs BR + bortezomib) → (R2 vs rituximab)	NCT01415752	II	Ongoing			
(BR + ibrutinib → rituximab + ibrutinib)	NCT01776840	III	Ongoing			
BR + acalabrutinib vs BR	NCT02972840	III	Ongoing			
BR + venetoclax	NCT03834688	II	Ongoing			
Bendamustine/obinutuzumab/venetoclax	NCT03872180	II	Ongoing			
Ibrutinib + rituximab	NCT01880567	II	49 [28]	98 [60]	NR	Myalgias (14), fatigue (14), dyspnea (10), a.fib (8)
R2	NCT01472562 [106, 107]	II	38 [64]^a^	92 [64]	NR	Infections (19.4), tumor flare (11), abdominal pain (5), serum sickness (5), syncope (5), neutropenic fever (5)
Acalabrutinib + R2	NCT03863184	II	ongoing			
Venetoclax + ibrutinib + obinutuzumab	NCT02558816^b^ [85]	I	15 [NP]	100 [47]	NP	Hepatobiliary disorder (27), rash (7)
Acalabrutinib + rituximab + (bendamustine or venetoclax)	NCT02717624	I	ongoing			

Abbreviations: *NR* not reached, *NP* not presented, *ORR* overall response rate, *CRR* complete response, *PFS* progression-free survival, *A.fib* atrial fibrillation, *R2* lenalidomide (Revlimid) and rituximab, *R-HAD* rituximab, cytarabine, dexamethasone

^a^Results are for both younger patients with low and intermediate MIPI scores and older patients with all MIPI scores; results for specific for older patients were not presented

^b^Step C of the OAsIs trial

*f/up in months

### Approaches for the unfit patient which include chemotherapy

In the Nordic MCL4 (LENA-BERIT) study, treatment naïve elderly patients (> 65) or younger patients unfit for chemotherapy were treated with BR in addition to lenalidomide for a total of 6 cycles followed by maintenance lenalidomide for a total of 7 cycles. The MTD of lenalidomide with BR was found to be 10 mg with lenalidomide started at the second cycle of treatment due to high rate toxicity when started concurrently with BR. In the study (*n* = 50), the ORR was 80% (CR rate = 64%) with 56% MRD negativity after BR-lenalidomide (and no increase in MRD negativity after maintenance therapy) [113]. Grade 3 or higher infections were found in 42% of patients with opportunistic infections in three patients (two cases of PJP and one case of CMV retinitis), likely due to the significant lymphosuppression from the bendamustine and lenalidomide combination. The high rate of infectious complications in this trial dampened enthusiasm for using this combination. R-CHOP with lenalidomide may have a more tolerable toxicity profile with less lymphosuppression and is currently under investigation (NCT02633137).

An ongoing phase III European R2 Elderly study is evaluating the benefit of lenalidomide given in the maintenance setting. This is a trial with a 2 × 2 factorial design in which patients are first randomized to either alternating cycles of R-CHOP and R-HAD (rituximab with high-dose ara-C and dexamethasone), each for 3 cycles versus 8 cycles of R-CHOP with each arm being further randomized to either maintenance rituximab or maintenance rituximab with lenalidomide for two years. ECOG 1411 is another phase III trial evaluating maintenance lenalidomide in older patients. This trial also has a 2 × 2 factorial design in which patients are initially randomized to BR with or without bortezomib followed by randomization of each arm to maintenance rituximab with lenalidomide versus rituximab monotherapy for two years. The results of both these studies will define the role of maintenance lenalidomide when given after different chemoimmunotherapy regimens in elderly patients with MCL.

Currently, the ibrutinib and acalabrutinib are both being evaluated in combination with BR in two phase III randomized trials. Unlike the combination of R-CHOP and ibrutinib which may be difficult to tolerate in the older patient as discussed previously, the combination of BR with ibrutinib was found to be safe with no dose-limiting toxicities in a phase 1/1b study of R/R NHL which included older patients [114]. In the study, the ORR was 94% (CR rate = 76%) in relapsed/refractory MCL patients (*n* = 17), thus demonstrating the potential efficacy of combined BTK inhibition with immunotherapy. The SHINE study (NCT01776840) will compare the combination of ibrutinib with BR followed by ibrutinib and rituximab (IR) maintenance versus BR + rituximab maintenance. There is another study (NCT02972840) looking at the combination of acalabrutinib and BR but will not include maintenance acalabrutinib.

Clinical trials with venetoclax incorporated into BR for the older population are also ongoing. An initial dose-finding study of venetoclax on a continuous schedule with BR in NHL of mixed histologies initially established a dosage of 800 mg given continuously to be safe [115]. However, a subsequent FL study that included three separate arms (venetoclax 800 mg and rituximab, venetoclax 800 mg + BR, and BR alone) demonstrated a high rate of grade 3–4 AE (78%) [116]. As both these trials enrolled both younger and older patients, this dosing was expected to lead to excessive toxicity in the older population. Thus, the ongoing phase II single-arm study (PrE0405) evaluating BR with venetoclax in patients 60 years and older is using dosing of 400 mg given 10 days starting on day 1 of BR [117]. Finally, the combination of venetoclax/bendamustine/obinutuzumab is currently being investigated for older patients in the upfront setting (NCT03872180).

### Chemotherapy free approaches for the transplant-ineligible patient

As discussed previously, IR has shown impressive activity in the upfront setting in younger patients [109]. Not surprisingly, this combination has also shown promising results for elderly untreated patients [118]. In a phase II study enrolling patients ≥ 65 with untreated MCL, patients received continuous IR with rituximab initially given monthly for 8 cycles followed by every 2 months until disease progression [118]. At a median follow-up of 28 months (*n* = 49), 61% were still on therapy with a relatively high number of patients discontinuing treatment due to atrial fibrillation 14%), although 22% of patients enrolled had a prior history of atrial fibrillation. Among the evaluable patients (*n* = 42) the ORR was 98% (CR rate = 60%) and median PFS was not reached. Thus, IR has high activity in the front line setting for elderly patients, although those with cardiac co-morbidities should be monitored closely.

R2 was studied in untreated young MCL patients with a low-risk disease with low/intermediate MIPI score as well as elderly transplant-ineligible patients [106, 107]. The elderly patients (> 60 years) comprised 63% of the study population. Although a subset analysis of older patients was not performed due to the small sample size, it is notable that at 5-year follow-up, patients with high MIPI scores (which only included older patients) had a similar PFS as low/intermediate-risk patients (*p* = 0.82). This suggests that R2 may be as active in the high-risk older patient as the low-risk younger patient [106]. R2 with acalabrutinib (ALR) is currently undergoing investigation in a phase II trial of untreated MCL which includes older patients (NCT03863184).

The combination of venetoclax/ibrutinib/obinutuzumab is currently undergoing evaluation in the OASIS study that is enrolling both relapsed/refractory and newly diagnosed MCL. Preliminary results of the newly diagnosed cohort (*n* = 15) have recently been reported [85]. Venetoclax was given at 400 mg continuously after a cycle 1 ramp-up phase. Five patients had non-hematologic grade 3–4 toxicity including elevated LFTs and rash, and two patients had grade 3–4 hematologic toxicity. Seven patients went on to complete 6 cycles, all were in CR with no MRD. A multi-institutional trial with the combination of acalabrutinib/venetoclax/rituximab in treatment naïve MCL (along with a separate arm with treatment naïve and relapsed/refractory MCL receiving BR and acalabrutinib) is ongoing (NCT02717624).

## Risk-adapted approaches in MCL—*are we there yet*?

A risk-adapted approach involves the evaluation of data obtained during interim assessments to make ongoing changes to the therapeutic plan to tailor the amount or type of therapy to achieve the most optimal long-term response with the least toxicity. It is different from typical clinical decision making in that specific pre-set rules are in place before starting any therapy to guide the clinician down a predetermined therapeutic path. The interim assessment can be imaging studies such as PET or the presence of MRD. MRD is defined as detectable cancer cells present after completion of therapy. These cells can be detectable with many different methods but most commonly by flow cytometry or polymerase chain reaction (PCR). Risk-adapted approaches incorporating MRD assessments have been firmly established in other hematologic malignancies, most notably chronic myelogenous leukemia (CML) and ALL, and is currently under investigation in MCL.

A recent study in the contemporary era showed that young patients (age ≤ 65) who received auto-HCT in CR1 had superior PFS than those who did not (75 versus 44 months, respectively) [119]. In this study, 76% of patients achieved a CR before proceeding to transplant. This group of patients still maintained a significant PFS benefit on subset analysis. A study looked at the prognostic significance of MRD status in patients who are in CR after induction while treated on two prospective European MCL clinical trials. In the study, the absence of MRD (as assessed by either flow cytometry or allele-specific oligonucleotide PCR (ASO-PCR) for IgH or cyclinD1 rearrangement) was shown to be associated with a more prolonged response duration compared to patients with MRD positivity in patients who achieved a CR after induction therapy (2-year response duration; 94% versus 71%) [120]. However, the potential benefit of transplant in MRD-negative patients could not be determined as all eligible young patients in this study went on to receive transplants.

Given the toxicities and resource utilization of auto-HCTs, patients in CR after induction chemotherapy with MRD negativity are an ideal group for a prospective risk-adapted study. The ECOG-ACRIN 4151 study is a phase III randomized study that is currently ongoing in which patients (ages 18–70) that achieve MRD negativity after undergoing induction chemotherapy (with regimen at the discretion of treating physician) will be randomized to either rituximab maintenance therapy or auto-HCT followed by maintenance rituximab. MRD assessment is from peripheral blood assessing for circulating tumor DNA (ctDNA) with immunoglobulin high throughput sequencing (Ig-HTS). The study is powered for OS at 6 years following induction therapy.

Another potential risk-adapted approach that has gained considerable attention recently is the measurement of ctDNA levels at regular intervals throughout the course of therapy rather than once after therapy. This approach may provide more sensitive and frequent therapy response assessment compared to interim PET or CT imaging and thus provide more opportunities for a change of therapy if the anticipated response is not achieved. This approach is analogous to response monitoring of CML to TKI therapy by PCR assessment of the peripheral blood for Bcr-Abl transcripts that is now standard of care. Two studies in MCL shown that achieving decreased ctDNA levels earlier during chemotherapy was associated with a better PFS [121, 122]. Many MCL trials using targeted therapies are incorporating MRD assessments into their protocols as secondary endpoints. This data will be valuable in creating future prospective studies exploring whether shorter courses of therapy could be given in patients who achieve MRD negativity without compromising long-term outcomes. In addition to response assessment, ctDNA testing can theoretically detect the presence of new mutations that predict therapy resistance potentially allowing a change of therapy sooner than when the progressive disease is detected. More prospective MRD based risk-adapted trials will likely be on the horizon, which will hopefully bring MRD analysis into regular clinical practice in MCL.

## Conclusions

### How will we approach MCL treatment in the future?

One of the hot topics in the field of MCL is whether chemo-free approaches using targeted agents will fully supplant chemotherapy in the upfront setting. Although ibrutinib and rituximab have shown good clinical activity in treatment naïve MCL patients and chemo-free combinatorial approaches (ibrutinib, venetoclax, and anti-CD20 therapy) have gained ground in the frontline treatment of CLL, it is still early days in the world of MCL to abandon chemotherapy in the frontline setting at this time. On the other hand, a combination of novel agents and chemotherapy appears promising. The SHINE and TRIANGLE studies will be able to provide a more definitive answer.

For the younger patient, a chemo-free approach may be an option for the low-risk patient (MIPI low/intermediate) although its role in more aggressive disease with high Ki-67 is less clear. Incorporating targeted agents to reduce the amount of chemotherapy needed to obtain the same durable response, such as in NCT02427620, maybe a more attractive approach. Incorporating MRD negativity prospectively to decide if further consolidative chemotherapy is necessary, as in ECOG 4151, would be valuable if targeted agents are used upfront.

For relapsed/refractory MCL, with the recent results of ZUMA-2, CART therapy will likely play a crucial role in this setting, most notably for patients who progress on BTK inhibitors where there are no therapies currently available to achieve a durable response. BiTe antibodies have shown promising results in heavily pre-treated relapsed/refractory MCL, including those who had previously received CART, which provides yet another option in the growing armamentarium of MCL therapeutics. However, their place in the MCL treatment paradigm will need to be better defined, particularly whether they should be used earlier on, perhaps after BTK inhibitor failure, or following the progression on CART.

With the large number of ongoing studies in MCL, it is no doubt a very exciting time in the field. Given the rarity of MCL compared to other B cell malignancies, it will take time for clinical trials to fully mature to change standards of care. Hence, it is extremely important to enroll patients on clinical trials. With time, the way we treat MCL is going to change significantly in the not so distant future with new approaches shaping multiple levels of treatment.

## Data Availability

Not applicable

## Abbreviations

Allo-HCT: Allogeneic hematopoietic stem cell transplantation
Auto-HCT: Autologous hematopoietic stem cell transplantation
BiTe: Bispecific T cell engager
BR: Bendamustine and rituximab
BTK: Bruton’s tyrosine kinase
CAR: Chimeric antigen receptor
CR: Complete response
MCL: Mantle cell lymphoma
MIPI: Mantle cell lymphoma international prognostic index
NHL: Non-Hodgkin’s lymphoma
ORR: Overall response rate
OS: Overall survival
PFS: Progression-free survival
R2: Lenalidomide (Revlimid) and rituximab
R-CHOP: Rituximab, cyclophosphamide, doxorubicin, vincristine, and prednisone
